# Can community midwives prevent antenatal depression? An external pilot study to test the feasibility of a cluster randomized controlled universal prevention trial

**DOI:** 10.1017/S003329171500183X

**Published:** 2015-10-20

**Authors:** T. S. Brugha, J. Smith, J. Austin, J. Bankart, M. Patterson, C. Lovett, Z. Morgan, C. J. Morrell, P. Slade

**Affiliations:** 1 Department of Health Sciences, Section of Adult Social and Epidemiological Psychiatry, University of Leicester, Leicester General Hospital, Gwendolen Road, Leicester, UK; 2 Division of Women's and Children's CMG, Leicester Royal Infirmary, Jarvis Building, Infirmary Square, Leicester, UK; 3 School of Health Sciences, University of Nottingham, Queen's Medical Centre, Nottingham, UK; 4 Clinical Psychology – Ground Floor Whelan Building, Institute of Psychology Health and Society, University of Liverpool, Liverpool, UK

**Keywords:** Depression, perinatal, pilot study, prevention, randomized controlled trial

## Abstract

**Background:**

Repeated epidemiological surveys show no decline in depression although uptake of treatments has grown. Universal depression prevention interventions are effective in schools but untested rigorously in adulthood. Selective prevention programmes have poor uptake. Universal interventions may be more acceptable during routine healthcare contacts for example antenatally. One study within routine postnatal healthcare suggested risk of postnatal depression could be reduced in non-depressed women from 11% to 8% by giving health visitors psychological intervention training. Feasibility and effectiveness in other settings, most notably antenatally, is unknown.

**Method:**

We conducted an external pilot study using a cluster trial design consisting of recruitment and enhanced psychological training of randomly selected clusters of community midwives (CMWs), recruitment of pregnant women of all levels of risk of depression, collection of baseline and outcome data prior to childbirth, allowing time for women ‘at increased risk’ to complete CMW-provided psychological support sessions.

**Results:**

Seventy-nine percent of eligible women approached agreed to take part. Two hundred and ninety-eight women in eight clusters participated and 186 termed ‘at low risk’ for depression, based on an Edinburgh Perinatal Depression Scale (EPDS) score of <12 at 12 weeks gestation, provided baseline and outcome data at 34 weeks gestation. All trial protocol procedures were shown to be feasible. Antenatal effect sizes in women ‘at low risk’ were similar to those previously demonstrated postnatally. Qualitative work confirmed the acceptability of the approach to CMWs and intervention group women.

**Conclusion:**

A fully powered trial testing universal prevention of depression in pregnancy is feasible, acceptable and worth undertaking.

## Introduction

Depression continues to be a leading cause of disability (Murray *et al.* 2012) worldwide: the Global Burden of Disease (GBD) studies underlined the ‘large unrecognized burden of mental illness in developed and developing countries – 8.5% of disability adjusted life years (DALYs) in the GBD 1990 study and 10.1% in the GBD 2000 study’. It drew attention to ‘the urgent need for identification and implementation of effective and affordable strategies for this set of problems’ (Vos *et al.* 2012). Randomized controlled trial (RCT) evidence shows that pharmacological and psychological interventions can be recommended for depression (NICE, 2009). However, despite this and evidence of increased uptake of depression treatments, epidemiological studies monitoring rates of depression at a population level show no evidence of decline in depression prevalence (Brugha *et al.* 2004; Kessler *et al.* 2005; Compton *et al.* 2006; Spiers *et al.* 2012). Although factors other than treatment may explain trends in depression rates, we argue innovative approaches to tackling depression (Dowrick & Frances, 2013) such as the use of prevention are needed.

Targeting older schoolchildren at high risk of becoming cases of depression with school-based psychological prevention programmes seems to be effective (Garber *et al.* 2009). However, prevention policies have largely failed to access high-risk adult populations (Cuijpers *et al.* 2010), because of stigma and lack of perceived relevance to potential users. Selected and indicated approaches, for example targeting high-risk groups, also face the limitation that the few who benefit are unlikely to alter significantly population prevalence and thus overall societal burden. It has been recommended to position prevention services in primary care or to integrate prevention interventions in community-wide interventions (Cuijpers *et al.* 2010).

Universal prevention (Mrazek & Haggerty, 1994) approaches involve people who may develop a condition in the future but not identifiably at risk currently. There has been hardly any evaluation of universal approaches to preventing depression in adulthood because of cost and the very large study sizes needed (Munoz *et al.* 2012) although small effects in large populations can have greater societal impact (Glasgow *et al.* 1999). Furthermore, this has not deterred child researchers from using this approach to randomize schools and classrooms and showing prevention of depression, albeit mainly in children at increased depression risk (Calear & Christensen, 2010).

Particular opportunities for prevention action could exist when people face challenging life transitions that offer frequent contact with experienced health professionals. For example during pregnancy and after child birth, there is both increased actual or perceived risk and normal access to non-stigmatizing care. Stigma and the social context of childbirth mitigate against active disclosure of emotional distress (Slade *et al.* 2010). Encouraging evidence that a psychologically focused reorganization of care, could be acceptable, feasible and effective comes from a cluster RCT of health visitor training for postnatal depression (Morrell *et al.* 2009). In women who at 6–8 weeks following childbirth were termed ‘at low risk’ of depression, based on a negative test on the Edinburgh Postnatal Depression Scale (EPDS; Cox & Holden, 1994), risk of depression was reduced from 11% to 8% at 6 months postnatally if their health visitor had been trained to offer additional psychological support (Brugha *et al.* 2011). Furthermore, the development of symptoms of depression was experimentally shown to be less likely where the health visitor had also evaluated and discussed 6–8 weeks after childbirth with the ‘at low risk’ mother her risk of depression (Brugha *et al.* 2011), although not providing therapy sessions unless indicated. These findings suggest a possible ‘knock-on’ or ‘trickle-down’ effect on non-depressed (i.e. ‘not at risk’) women of additional psychological evaluation and intervention skills training of their health visitor, which was originally intended to benefit only depressed (i.e. ‘at risk’) women postnatally.

The greater risk of depression in women (compared to men) appears to begin after the menarche, continues throughout the childbearing years, diminishes following the menopause and is higher in married and cohabiting women (Bebbington *et al.* 2003; Angold & Costello, 2006; Seedat *et al.* 2009). Depression may impact adversely on fetal growth and development (particularly in males) (Davalos *et al.* 2012). Depression in pregnancy strongly predicts depression postnatally, which links to problems in the mother–infant relationship and attachment (Evans *et al.* 2001). Even when there are no medical complications women in most parts of the developed world have frequent contact with a trained health practitioner in pregnancy. In the UK antenatal care is provided primarily by a community midwife (CMW) who will typically see a woman throughout her pregnancy on at least 10 occasions if they are primigravida (seven occasions if multigravida) [National Collaborating Centre for Mental Health (Great Britain) & National Institute for Health and Clinical Excellence (Great Britain), 2008]. Nationally, few midwives have specific training in psychological care and many have identified areas of practice they would wish to improve before taking on such a role (Stewart & Henshaw, 2002). However, we do not know whether additional psychological training could be provided to and used effectively by CMW staff working in universally provided antenatal clinics. Before mounting a perinatal depression prevention trial to test the possible ‘knock-on’ benefits for women ‘not at risk’ of depression a feasibility pilot trial involving CMWs caring for women antenatally and given such additional psychological training was needed.

### Aim

In an external pilot study (Lancaster *et al.* 2004) our aim was to assess the feasibility and acceptability to pregnant women ‘not at risk’ of depression, and to carers and midwives, of training of CMWs in psychological approaches to prevent the development of depression in pregnancy compared with usual care provided by CMWs with no additional training.

The external pilot study objectives (Lancaster *et al.* 2004) included determining acceptability of procedures for selecting and randomizing CMW clusters; intervention training and implementation; recruitment rates of pregnant women, including women with few or no symptoms of depression; whether intervention group-trained CMWs could undertake an assessment of depressive symptoms on all women under their care as part of routine antenatal care; and for women at increased risk of depression whether CMWs could provide acceptable psychological support sessions. Additional objectives were outcome data collection at 34 weeks gestation; generation of estimates of the variability of outcome measures; estimation of full trial resourcing; qualitative exploration of the acceptability to women of CMWs providing specific emotional care and to explore CMWs' perceptions of training and changes to practice; quantitative measurement of relationship quality between women and their CMW, with the potential to explain possible future prevention benefit mechanisms underlying such interventions.

## Method

The Pregnancy and Wellbeing external pilot Study (PAWs; Brugha *et al.* 2012) employed a cluster randomized controlled (C-RCT) design in which clusters of CMWs were the unit of randomization. The study was primarily on women assessed at study entry at 12 weeks gestation as not at increased risk of depression. Quantitative outcomes were collected by post or online (depending on the woman's choice) at 34 weeks gestation. Primary quantitative outcome: proportion of EPDS (Cox & Holden, 1994) negative women (EPDS < 12, low risk women) at 12 weeks gestation who were EPDS positive (EPDS ⩾ 12) at 34 weeks.

Ethical and research governance approvals and trial registration (ISRCTN72346869) were obtained.

### Procedures

**Fig. 1.**
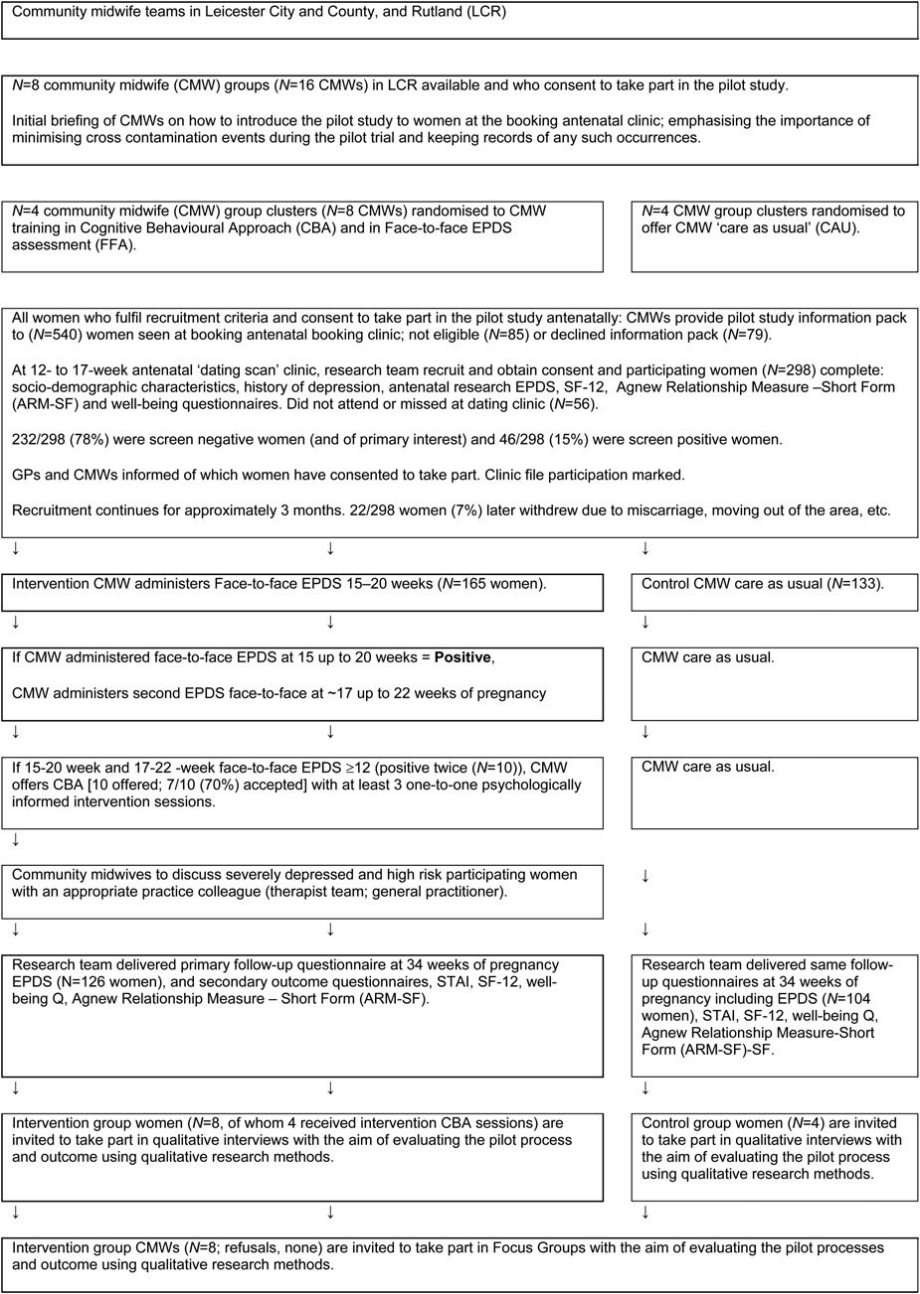
Recruitment and follow-up of women antenatally.

Eight CMW clusters (each consisting of at least two CMWs who worked in the same practice setting) were randomized, four clusters to the intervention group (IG) and four to care as usual (CAU) (Fig. 1). IG CMWs received 8 days of training (Morrell *et al.* 2011) by the same trainers that took part in the PoNDER RCT, adjusted to fit the context of pregnancy. Women cared for by trial-participating CMWs (IG and CAU) antenatally were invited to consent to take part and to complete baseline measurements.

### Randomization

CMW group/cluster random allocation was stratified by local authority (Leicester City *v.* the two county local authority areas combined into one county stratum) due to the marked urban–rural differences in the socioeconomic composition of these populations. The random sequence allocation schedule was prepared and undertaken by an independent statistician, blind to the identity of the CMW cluster groups. Randomization software was used to generate the random allocation sequence for the CMW groups/clusters (random permuted blocks of variable size stratified by size of CMW cluster delivery rate provided by the CMW service). Each cluster comprised two CMWs: there were four clusters of intervention CMWs and four clusters of CMWs that provided CAU.

### Recruitment of pregnant women

Women were recruited from local primary-care practices according to which CMW provided their antenatal care. Participating IG and CAU CMWs at booking antenatal clinics handed out study information packs to all women meeting eligibility criteria. At 12 weeks gestation women who had not refused to take part were approached by research staff blind to IG and CAU allocation to obtain informed consent and collect baseline data. Inclusion criteria were: booked by the 18th week of pregnancy, at least 18 years of age, able to give informed consent, residing in the UK and intending to remain there 6 months after the birth of the baby, able to read and fully comprehend English. Exclusion criteria were: not able to give informed consent for any reason, not a resident of the UK, or would not remain in the UK for 6 months after the birth of the baby, unable to fully comprehend and read English, in receipt of treatment from specialist mental health services and women presenting later than 18 weeks of pregnancy to the maternity services.

### Masking

Recruiting research staff were blind to IG CAU status. When CMWs consented to take part they were blind to allocation, made aware of it before training and therefore aware of which women under their care were participating in the pilot. CMWs and research staff were blind to research data collected. Participating women could not be blinded to the intervention allocation.

### Intervention

Cluster level training (Fig. 1) of IG CMWs was adapted for antenatal care and was otherwise identical to that used previously to train health visitors (Morrell *et al.* 2011).

Training for the individual-level intervention was 1 day on assessment of depressive symptoms in pregnancy and in the use of the EPDS, and 7 days of training in psychological care based on a cognitive behavioural approach (CBA).

Training focused primarily on depression, including ‘face to face assessment’ (FFA) of symptoms of depression including use of the EPDS (Cox & Holden, 1994) by the participating women, and also acknowledged the frequent co-existence of symptoms of general anxiety. CBA training focused on describing key features of therapeutic relationships; facilitating formation, development and maintenance of therapeutic relationships, conducting assessments of clients with depression during pregnancy using a Five Areas approach followed by implementation of a range of simple cognitive behavioural approaches as appropriate (Morrell *et al.* 2011). The five areas are: ‘the environment (life situation/relationships and practical problems), cognitions (altered thinking), emotions (altered mood), physiology (altered physical symptoms), and behaviour (altered behaviour). […] The approaches were placed in the context of a collaborative therapeutic relationship between the health visitor and the client and there was little emphasis in the training on the theoretical or research underpinnings of the approaches being utilized.’ IG CMWs were given active feedback and guidance on practice in the use of CBA by their trainer via two half-days of reflective practice and subsequent 4–6 weekly group supervision slots. CMWs could also access support by telephone and email contact with a psychological therapy practitioner regarding implementation of psychological care.

Although any IG woman who scored ⩾12 on the EPDS at the 14th and 16th week antenatal CMW clinic visit was not of primary interest to the study (Fig. 1) she was offered up to three CBA sessions by her IG CMW (thus ensuring that midwives put their CBA training into practice).

### Measures

At baseline (week 12 of gestation) all women completed self-report questionnaire measures, repeated at 34 weeks of pregnancy: socio-demographic details, the EPDS (Cox & Holden, 1994), state anxiety with the State-Trait Anxiety Inventory (STAI; Spielberger *et al.* 1983), the Agnew Relationship Scale – short form (ARM-12; Agnew-Davies *et al.* 1998) and Satisfaction with Life Scale (SWL; Diener *et al.* 1985). The EPDS has been validated in pregnancy (Murray & Cox, 1990; Cox *et al.* 1996) and is also termed ‘Edinburgh Perinatal Depression Scale’ when used during pregnancy (Cox *et al.* 2014). All baseline measures were completed on paper; women could choose postal or online completion (triggered by email alerts) of outcome questionnaires (34 weeks). IG and CAU CMWs collected service-use contact data by the participating women which would be needed for health economic analyses.

### Analysis

As the study was an external pilot, the main aim of which was to collect process outcomes related to feasibility, there was no formal sample size calculation and no formal hypothesis testing or group comparison (Lancaster *et al.* 2004) was performed on the quantitative data. We judged 300 women would be needed to evaluate a full training group of eight CMWs, test sufficiently the assessment (FFA) and intervention (CBA) pilot components and provide sufficient qualitative observational data. Descriptive statistics of the baseline characteristics and follow-up measures were produced, and multi-level models were run to allow for the clustered nature of the data, although purely in order to generate estimates of the variability of outcome measures (with adjusted s.e.s). These estimates were generated in SAS (SAS Institute Inc., 2012). The pilot was reported according to the CONSORT guidelines for cluster trials (Campbell *et al.* 2004, 2012).

### Qualitative data collection and analysis

A stratified subsample of 26 intervention group women [all those with EPDS scores ⩾12 at baseline (*N* = 7) and a random sample of low-scoring women (*N* = 19) representing the full range of EPDS scores and parity (further details available on request)], having completed the 34-week pilot outcome, were invited to take part in a qualitative evaluation of the pilot. C.L., closely supervised by an experienced qualitative researcher (P.S.), conducted the face-to-face interviews. The main focus was to understand women's perspectives on CMWs assessing the presence of depressive symptoms in pregnancy and the offer of psychologically informed input within the routine CMW care context.

A ‘template’ approach to qualitative data analysis was used (King, 1998). This is a useful hybrid approach allowing a pre-specified template of themes that are particularly important to the research, to be applied to address particular questions, while allowing further, richer detail in the form of additional themes or subthemes to emerge from the data. It contrasts with purely exploratory methods, which would not have been appropriate here, as there were some specific questions to address. Templates are made up of codes that are hierarchically organized: the highest-level codes are the broad themes, while lower-level codes are more narrowly focused aspects of the broader theme. The original pre-specified template focused on the main research questions which included women's perspectives on the quality of their emotional care from their CMW, their views on the use of the assessment of depressive symptoms and their experience of and views about CMW-based emotional care and support.

All eight intervention CMWs were invited by anonymous postal questionnaire to evaluate the training both immediately after training completion and 6 months after implementation of trial-specified practice changes. Their written comments were subject to basic content analysis and rating scale results were analysed descriptively because of small size (*N* = 7). At the completion of the pilot, all eight CMWs were invited and attended a focus group giving their perspectives on their experiences of being trained and implementing psychological assessment and interventions in practice, led by P.S., audio-recorded and transcribed. A content analysis was completed according to recommended procedures to identify themes (Vaughn *et al.* 1996). Issues were identified in terms of initial codes and then combined to form higher codes reflecting consistent perspectives within the group providing insights into the identified areas of interest. Where there were disparate views these were specifically reflected in the analysis. A second coder (C.L.) reviewed the analysis: there was 97.4% agreement of allocation of comments to themes. Of 233 statements six were resolved through discussion. The themes were also presented back to focus group members for comment as a part of the validation process. The feedback was that they were felt to reflect the views as expressed. No omissions or perceived inaccuracies were noted.

## Results

All essential, core pilot procedures and stages were successfully carried out (Brugha *et al.* 2012). Participating CMWs successfully completed training and used all protocol-specified procedures throughout the pilot. Of 540 potentially eligible women attending clinics, 85 were not eligible (language difficulties, about to leave the area, etc.); 40 who attended the recruitment clinic were missed and 16 did not attend, 22 were later excluded (miscarriage, failure to collect data, etc.), leaving 377 eligible to take part of whom 79 were asked but refused to be recruited (79/377, 21% refusals). Thus 298/377 (79%) of women approached were recruited. Sociodemographic characteristics of the 298 eligible women who agreed to take part are given in Table 1 showing women followed up and lost to follow-up, compared to a random sample of other women booking at antenatal clinics. Black and ethnic minority women appeared less likely to take part, to be followed up and to speak English as a first language. Women lost to follow-up were more likely to be living with ‘others’ and to be unemployed (Table 1, Fig. 1). Eighty (27%) of 298 women enrolled had past depression: 39/165 (24%) of IG women and 41/133 (31%) of CAU women. Of those who had a history of depression 19/39 (49%) were randomized to the IG and 21/41 (51%) to the CAU group (no real difference). Of 298 recruited women, 232/298 (78%) were ‘screen negative’ women and 46/299 (15%) were ‘screen positive’. Seventy-two women (24%) initially requested a follow-up questionnaire by post; 226/298 (76%) requested an online version of whom 70/226 (31%) later asked to change to a postal version. Detailed lessons gained and further detailed recommendations for running a full-scale trial across different sites are set out in the full project report (Brugha *et al.* 2012), available on request.

**Table 1.** Baseline demographic data

	Recruited women followed up (*N* = 229)	Recruited women lost to follow-up (*N* = 69)	Recruited women (total = 298)	Population reference group (total = 1012)
English as first language	201 (87.8%)	53 (76.8%)	254 (85.2%)	699 (69.1%)
Living with a partner	206 (90.0%)	46 (66.7%)	252 (84.6%)	800 (79.1%)
Living with others	16 (7.0%)	16 (23.2%)	32 (10.7%)	52 (5.1%)
Previous depression	63 (27.5%)	17 (24.6%)	80 (26.9%)	172 (17.0%)
First baby	107 (46.7%)	25 (36.2%)	132 (44.3%)	418 (41.3%)
Working – mother	177 (77.3%)	39 (56.5%)	216 (72.5%)	636 (62.8%)
Looking after the home – mother	40 (17.5%)	15 (21.7%)	55 (18.5%)	206 (20.4%)
Unemployed – mother	6 (2.6%)	8 (11.6%)	14 (4.7%)	76 (7.5%)
Partner working	192 (83.8%)	40 (58.0%)	232 (77.9%)	809 (79.9%)
Looking after home – partner	12 (5.2%)	2 (2.9%)	14 (4.7%)	2 (0.2%)
Unemployed – partner	9 (3.9%)	9 (13.0%)	18 (6.0%)	62 (6.1%)
Smoker – mother	20 (8.7%)	12 (17.4%)	32 (10.7%)	164 (16.2%)
Ethnic origin				
White British	191 (83.4%)	49 (71.0%)	240 (80.5%)	686 (67.8%)
Asian Indian	17 (7.4%)	4 (5.8%)	21 (7.1%)	139 (13.7%)
Asian Pakistani	2 (0.9%)	0 (0.0%)	2 (0.7%)	23 (2.3%)
Black African	2 (0.9%)	1 (1.4%)	3 (1.0%)	36 (3.6%)
Other	17 (7.4%)	15 (21.7%)	32 (10.7%)	32 (3.2%)

Recruited women, showing followed up and lost to follow-up, compared to a population reference group, based on a sample of 1012 non-recruited women taken at random from 8/9 week booking appointments.

The EPDS scores collected at 34 weeks (which were not powered to identify statistically significant differences) are given in Table 2. There were 7.8% of IG women and 10.8% of CAU women at low risk who were EPDS positive at 34 weeks gestation. The percentages for all women including those at high risk (EPDS positive at 12th week gestation) were 11.1% and 19.4%, respectively. Mean scores for the EPDS and other secondary outcomes are given in Table 3 (also not powered to identify statistically significant differences). ARM scores in women at low risk and in all IG and CAU women appeared to be very similar (Table 3). But in women at high risk the IG mean score was 68.41 (s.e. = 2.77) and the control mean was 65.12 (s.e. = 3.54) (higher scores are better), the high-risk IG group women having been offered CBA sessions. Participant log information on service-use contact was often not returned; systematic procedures would be needed to ensure completeness in a trial, using regular reminders.

**Table 2.** Proportion (primary outcome) and percentage EPDS positive at outcome: low risk women and all women

Outcome	Number of women with raised EPDS score (⩾12) at 34 weeks pregnancy	Proportion (%) of women with raised EPDS score (⩾12) at 34 weeks
Primary		
Proportion of low risk women (EPDS < 12) at 12 weeks who were EPDS positive (EPDS ⩾12) at 34 weeks		
Intervention	8/103	7.8%
Control	9/83	10.8%
Secondary		
Proportion of *all* women with EPDS score at 34 weeks who were EPDS positive (EPDS ⩾12)		
Intervention	14/126	11.1%
Control	20/103	19.4%

EPDS, Edinburgh Perinatal Depression Scale: lower score indicates fewer symptoms of depression.

**Table 3.** Secondary outcomes: mean scores on EPDS, STAI, SWLS at 34 weeks of pregnancy

Outcome	Women (risk level at 12 weeks of pregnancy)	Group	*N*	Mean	s.e.
EPDS score					
	Low risk (EPDS < 12)	Intervention	103	5.8	0.43
		Control	83	6.5	0.48
	High risk (EPDS ⩾ 12)	Intervention	21	11.1	0.83
		Control	15	12.2	0.98
	All women	Intervention	126	6.81	0.43
		Control	103	7.62	0.49
STAI	All women	Intervention	118	38.2	0.94
		Control	94	40.3	1.04
SWLS	All women	Intervention	129	28.6	1.08
		Control	104	28.8	1.08
ARM score (high score = better)					
	Low risk (EPDS < 12)	Intervention	98	71.74	1.13
		Control	82	72.61	1.25
	All women	Intervention	122	71.22	1.02
		Control	100	71.47	1.13

EPDS, Edinburgh Perinatal Depression Scale, lower score indicates fewer symptoms of depression; STAI, State Trait Anxiety Inventory; SWLS, Satisfaction with Life Scale; ARM, Agnew Relationship Measure (high score is better).

Seven women who completed the EPDS at 34 weeks, did not complete one at 12 weeks and therefore the totals for ‘All women’ do not match those of the ‘Low risk’ and ‘High risk’ combined.

### Women's perspectives (Table 4)

Most women perceived their CMW as being caring and supportive and appreciated their openness. Women strongly valued the CMW exploring and sharing how they were feeling. They welcomed the availability of support and the majority felt that CMWs were easy to talk to [statement 1 (s1)].

A small number of women said that they had not felt the need to share although, of these, most said they felt that they could have if needed. Where women felt they would not have been able to share their feelings, it was attributed to the fact that they had not built a relationship (s2).

The majority of women felt positive about CMWs using the EPDS and that this was in keeping with their role. Women generally felt that it was important to consider emotional as well as physical health and they valued the availability of support. Phrases used to express their feelings on the EPDS included ‘really good’, ‘potentially helpful’, ‘important as emotions do fluctuate’, ‘safeguards’ and ‘balances the views of women that care is all physical’ (s3). Two stated that they felt put ‘on the spot’ and another that she was ‘anxious about being called back’. A few women said that they found it difficult as they did not generally find it easy to discuss emotions (s4). Of the low-risk women who had not needed the sessions, most valued the availability of support for emotional wellbeing if needed. One woman offered psychologically informed sessions by the CMW (s5) commented that two home visit session were sufficient for her needs (altogether seven of ten women offered CBA sessions accepted (Fig. 1); one declined as she felt it was not required, as the low mood was due to a relative's illness; another women accepted and then declined and the third did not give a reason other than she felt it was not needed).

**Table 4.** Women's and community midwives’ (CMWs) perspectives based on qualitative interviews and questionnaires

(***a*) Womens' perspectives on emotional care provided by the CMW**	
The following statements illustrate this:	
s1	*‘And I was able to talk to her about the fact that I was feeling quite upset about other things and so she was able to talk me through, you know, just to reassure me that just because I'm feeling very emotional … does not affect the baby, and that kind of thing. So when I needed her to reassure me she did.’*
	*‘Whenever I've seen her if I have had anything I'm worried about I've told her and she's either told me what to do or said there's nothing to worry about or she's been very good yeah so I'm pleased about that.’*
	*‘I've felt that I can, you know, speak to her about anything.’*
A small number of women said that they had not felt the need to share, as illustrated by the following statement:	
s2	*‘I feel rushed each time … I feel like, it's literally “have you got your urine sample? I'll do your blood pressure. Anything else?” and literally pushed out the door.’*
CMWs carry out a specific emotional assessment	
The majority of women felt positive about CMWs using the Edinburgh Perinatal Depression Scale (EPDS) and that this was in keeping with their role. Phrases used to express their feelings on the EPDS included ‘really good’, ‘potentially helpful’, ‘important as emotions do fluctuate’, ‘safeguards’ and ‘balances the views of women that care is all physical’:	
s3	*‘They really highlighted how I was feeling and that I'd had a bit of a rough time …’*
	*‘I enjoyed it ‘cos it asked me questions that I didn't realize about myself, ‘cos you just think “oh, it's just another day” and then when you read it you're like “oh, ok, it's not just another day, I am feeling like that”.’*
A few women said that they found it difficult as they didn't generally find it easy to discuss emotions and there were some concerns about how honest women would be if they were feeling depressed or if their partner was present when they completed it:	
s4	*‘I mean my partner was with me at that appointment so, think sometimes you might have to do it on your own if you're gonna be 100% [honest].’*
Thoughts and feelings about the option of being offered psychologically informed sessions by the CMW.	
s5	*‘[midwife] was really forthcoming to say “I'll come and visit you at home, let's, you know, meet up, make a plan” and then she set me goals and I followed those through then she came back the week after and, you know, that was enough to sort me out and put me back on … the right path really.’*
**(*b*) Community midwifery perspectives**	
Focus group perspectives on the research implementation into the midwifery role and service:	
s6	*‘I think it's only recently, perhaps over the last couple of years that we've been able to identify people who might have depression, because of the score, is it the Whooley score that they use in the notes. We never used to have anything, but that's there now. We've not had any training on what to do with that, although we ask those questions.’*
The CMWs thought their training for using the EPDS was helpful but had some criticisms about aspects of the instrument. Using the EPDS face to face was seen as most useful but time was needed to be allocated to allow this.	
s7	*‘I think it's really good to have those skills that we've learnt. And I think it's probably going to be essential for all midwives to have them.’*
	*‘We've got this extra tool.’*
There were some aspects of the training that CMWs found frustrating. In particular the difference in pace with psychological work being slower than their typical approach was reflected in the training.	
s8	*‘It's so much slower. You're like, “come on; let's get to the meaty bit”. You know, we're very much like, we're on the go, we want to – that bit we've got now, what's the next bit?’*
Almost all CMWs reported applying the newly learnt approaches across a range of clients not just for the research.	
s9	*‘I found that it's built on perhaps what I was already using, and now where I have picked up ladies I can offer them a cognitive behavioural approach, that I feel is really useful and helpful to the women and it has filled that gap. I've been actually able to help some, and it's prevented them needing further help through the GP.’*
	*‘I think it has enhanced our skills to keep them [the women] well and to try and help them with problems that they have, even if it's not directly using it [with reference to other women not in the trial].’*
For the specific implementation it was important that time promised by managers was provided and despite assurances this was not always felt to be the case. There were different views as to whether all or some CMWs should be trained or whether this should be a routine part of student midwifery training.	
s10	*‘Well, I think that everyone should have the training because then you're just using that approach without even realizing it and I think that's what's going to help in the future – you haven't got to have a specialist counsellor, and have this expensive appointment that you're waiting for, to go see the counsellor, because you're using counselling skills.’*
	*‘As part of the normal student training, but I'm not convinced that it's a worthwhile thing for every – well, I don't know how you would do it, to give everybody that level of intensity and training that we've had.’*
In terms of supervision CMWs had felt well supported by the trainers and this support to implement the training was crucial. It needed to be provided by specialists and from outside the maternity service.	
s11	*‘But you need to have it; you need to know that it's there, to do this sort of thing.’*
CMWs felt all women should have equivalent access to this intervention. There were also comments about widening inclusion criteria and improving orientation to and communication within the research.	

### Community midwifery perspectives (Table 4)

Seven of eight CMWs completed the feedback questionnaires on the training. The training was well received and was seen as at an appropriate level for the midwifery role. CMWs rated their understanding and confidence in their skills to apply the assessment and CBA approach as good following the training but prior to implementation.

All intervention CMWs attended the focus group. They felt they were well positioned in the service structure to take on the emotional care of women in terms of how women viewed them, the personal nature of the care they provided and their accessibility. Midwives are expected to ask about emotions, under normal circumstances but (prior to the study), they had not been provided with training [Table 4 (s6)]. CMWs felt that using the EPDS was an important way of ‘flagging up’ to women that the CMW was interested in her emotional care. The cognitive behavioural training was viewed very positively, the skills were seen as useful and complementary to existing expertise. Perspectives on having developed the CBA skills were overwhelmingly positive (s7). Interestingly, almost all CMWs reported applying the newly learnt approaches across a range of clients not just for the research (s9). CMWs felt *all* women should have equivalent access to this intervention.

## Discussion

This study has provided valuable information on recruitment and participation rates, feasibility, and resources required to carry out a future multi-centre trial. Although interpretation of group comparisons in a feasibility study must be done with caution, quantitative outcomes collected at 34 weeks suggest that the approach shows promise in producing similar findings to those in the analysis of the lower risk women in the PoNDER trial (Brugha *et al.* 2011). Qualitative findings indicated that the trial procedures and intervention were acceptable to and welcomed by women, and that CMWs welcomed the training and the additional skills it provided for what was a clearly perceived health need that fits with their role, complements existing skills and addresses a gap in their training where they are expected to provide input but currently have neither the time nor expertise.

In the PoNDER postnatal RCT data analysis of lower risk women (Brugha *et al.* 2011) 83 (10.8%) out of 767 CAU women and 113 (7.7%) of 1474 IG women scored <12 on the EPDS at 6 months follow-up, an absolute difference of 3.1% [95% confidence interval (CI) 0.4–5.5] or an odds ratio (OR) of 0.68 (95% CI 0.50–0.93, *p* = 0.016). In the present external pilot study, at 34 weeks gestation (outcome), nine (10.8%) out of 83 CAU women and eight (7.8%) out of 103 IG women had an EPDS score <12. Therefore a planned fully powered trial is needed to determine whether a significant difference of such a magnitude would occur beyond chance during pregnancy. These pilot findings augment the case for conducting such a planned trial. These effect size estimates are also in line with formally synthesized findings in prevention of depression trials, albeit mostly using selected trial designs (Munoz *et al.* 2012) in which only persons at high risk of depression are included, not universal (unselected) samples as here.

Although black and ethnic minority women appeared less likely to take part, compared to a reference group of women using the same service, probably due to the requirement to fully comprehend English (Table 1), 298/377 (79%) of eligible women agreed to take part when approached. In our earlier postnatal trial (Morrell *et al.* 2009), 4084 (53%) of 7649 eligible women took part, which suggests that participation rates are higher when women are recruited antenatally. In The Netherlands about 750 000 working-age adults suffer from subthreshold depression each year, but the total number of participants in the widely advertised and free Coping with Depression (CWD) prevention courses is about 1% of this group (Cuijpers *et al.* 2010). The approach developed and implemented here, where the midwife is the active agent of prevention and potentially intervention, represents a breakthrough in overcoming population resistance to participation in depression prevention services (Cuijpers *et al.* 2010), which is also potentially a substantial step forward from generic awareness and signposting training (Department of Health, 2014).

Based on qualitative interview data, women clearly wanted good emotional support as a part of their routine care from CMWs, valued the opportunity to share how they felt and that emotional care would be there if needed. The specific emotional assessment was viewed positively with the main caveat that it was only early in pregnancy and might miss later distress. The availability of specific emotional assessment and care from the CMW was seen as important whether or not a woman herself needed specific input. A common element was that a confiding relationship indicated support was at hand if they should need it and this seemed to be important to and was appreciated by women at low risk. While the Agnew Relationship measure (ARM), originally developed for use following psychological therapy sessions, suggested differences only in the high-risk women, potential support availability, as needed, is flagged to all women. This information could be used to identify a set of quantitative questions for a future trial to assess low-risk women's relationships and perception of *availability* of support from their CMWs. This could identify a potential mechanism for any preventive effect (Brugha *et al.* 2011) in addition to evaluating the quality of alliance as in the low-risk sample actual contact in relation to emotional issues was limited.

The number of CMW teams (clusters) available to take part (approximately 20 clusters at the time of cluster recruitment) was not sufficient to conclude that a full trial evaluation could be carried out in one centre within reasonable time limits such as 2–3 years; a full trial would require collaboration with other centres.

As the proposed intervention requires only eight additional days training of existing staff and no costly additions to existing services as in current policies (Layard, 2006; Department of Health, 2014), providers and commissioners may feel emboldened to implement these findings in the absence of planned trial evaluation evidence. In that event we would urge the incorporation of experimental randomized ordering of staff cluster selection and training scheduling, together with routine collection of pre- and post-contact standardized depression measurement outcomes, in order that effectiveness can be objectively measured at relatively little extra cost.

This pilot study clearly demonstrates the feasibility of conducting a full-scale trial evaluation in this markedly neglected field of prevention research.
